# What’s new in Emergencies Trauma and Shock? Still searching for a scoring system for sepsis!

**DOI:** 10.4103/0974-2700.70740

**Published:** 2010

**Authors:** Michael D Grossman

**Affiliations:** Trauma, Surgical Critical Care and Emergency Surgery, St Lukes Health Network, Bethlehem, PA

Severity of illness scoring systems for critically ill and injured patients have been in existence for more than 20 years.[1] These tools were envisioned as a means of stratifying severity of illness among ICU patients in order to compare performance and outcomes. They use mortality as the discriminate end-point and have been utilized for ICU performance assessment, resource allocation, and clinical research. Most of those in common use including APACHE II and III, SAPS II, and MPM I and II, are based upon a series of physiologic variables coupled with chronic disease components and patient age. The ability of any such scoring system to predict mortality (or any other outcome) requires statistical analysis of an initial clinical database followed by validation of the model in the same data set or another population of patients. The most common method of assessing the validity of predictive models plots the relationship between true and false positive predictions for all predicted probabilities of the outcome in question and calculates the area under the curve (receiver-operator curve) as the C-statistic. A C-statistic in excess of 0.90 indicates a high proportion of true-positive predictions for any given calculated risk of the outcome. The ICU mortality prediction models noted previously have C-statistics between 0.83 and 0.90.[2]

In this issue of JETs Crowe and co-workers attempt to evaluate the effectiveness of three scoring systems developed for predicting hospital mortality based upon data available in the Emergency Department (ED).[3] These scores were evaluated for a series ED patients with severe sepsis and septic shock who received early goal-directed therapy (EGDT) and for whom observed in-hospital mortality was 32.9%. They suggest that these tools might be useful to stratify patients in order to define subsets that would benefit from treatment and that the specific scoring systems tested have the advantage of simplicity compared with more cumbersome and complex ICU scoring systems. In this study the authors found C-statistics between 0.59 and 0.74 with the MEDS score performing best. The authors conclude that these scores had relatively low validity in the current data set and should not be used to make decisions about patient care.

While these conclusions are supported by the authors’ data there are other important considerations implied by this work. Age, malignancy, and certain other variables common to all scoring systems might profoundly impact decisions to continue aggressive management following the ED phase of care. The frequency with which care is withdrawn as a byproduct of patient predetermination and/or failure to respond to resuscitative measures following the ED phase of care is often an unknown, as it was in the current study. Next, the idea that *hospital* mortality is equated with therapy provided in the ED is an important concept that has received only indirect analysis. Nonetheless, despite the myriad factors that potentially impact on outcome from severe sepsis following the ED phase of care, there appears to be a direct correlation between the presence of *early* and appropriate care and hospital mortality.[4] These observations also seem to hold true for the management of ST segment elevation myocardial infarction and thrombo-embolic stroke.[5,6] Perhaps lessons learned 40 years ago in the earliest trauma outcome studies describing a “golden hour” for trauma patients might be generalized to patients with critical illness.[7] Indeed commonly used scoring systems for predicting mortality including those used in the present study generally lack a “time since onset” variable and efforts to improve validity of ICU scoring systems have focused upon elements of care prior to arrival in the ICU (i.e. direct admission vs. transfer from a general care ward). Finally, given these assumptions regarding timely and aggressive care in the absence of patient pre-determination, the concept of scoring patients during the emergency phase of care might be somewhat counter intuitive. The sickest patients, most at risk of dying, would seem to be those most in need of early aggressive care.

In general, scoring systems in both critical care and emergency medicine may be more useful for research and quality improvement than clinical care. They do afford the opportunity to stratify and compare interventions, outcomes. and cost-effectiveness and in this sense are very useful in the administration of programs caring for acutely injured and ill patients. It seems however that repeated efforts to use these scores to determine which patients should be treated or when they should be treated are unlikely to be fruitful given the uncertainties of the scoring systems when applied to individual patients.

It has now been well established that the timely care of acutely ill and injured patients using basic principles of resuscitation offers the best opportunity for a favorable outcome. Current iterations of ICU scoring systems include measures of the *response* to these interventions. Since the capacity to reverse physiologic abnormalities and the time course over which cellular homeostasis is restored seems to be the best determinant of observed outcome, it seems unlikely that any of the currently available scoring systems provide adequate sensitivity to make decisions about whether or not to initiate aggressive measures during the early resuscitative phase of care in the ED. In short, these systems provide too little discrimination too soon in the continuum of care.
